# CE Separation and ICP-MS Detection of Gold Nanoparticles and Their Protein Conjugates

**DOI:** 10.1007/s10337-017-3387-y

**Published:** 2017-08-24

**Authors:** Joanna Legat, Magdalena Matczuk, Andrei Timerbaev, Maciej Jarosz

**Affiliations:** 1 Chair of Analytical Chemistry, Faculty of Chemistry, Warsaw University of Technology, Noakowskiego St. 3, 00-664 Warsaw, Poland; 2 Vernadsky Institute of Geochemistry and Analytical Chemistry, Kosygin St. 19, 119991 Moscow, Russian Federation

**Keywords:** Capillary electrophoresis, Mass spectrometry, Gold nanoparticles, Serum proteins

## Abstract

A full understanding and mediation of nanoparticle–serum protein interactions is key to design nanoparticles with vivid functions within the body, and to solve this problem one needs to differentiate and characterize individual nano-protein conjugates. In this paper, the authors applied capillary electrophoresis combined with inductively coupled plasma mass spectrometry detection to study the behavior of gold nanoparticles of different geometry, size and surface functionalization upon interacting with serum proteins and their mixtures. Due to high-resolution and -sensitivity benefits of this combined technique baseline separations were attained for free nanoparticles (at real-life doses) and different protein conjugates, and the conversion into the protein-bound form was scrutinized in terms of reaction time.

**Electronic supplementary material:**

The online version of this article (doi:10.1007/s10337-017-3387-y) contains supplementary material, which is available to authorized users.

## Introduction

Gold nanoparticles (AuNPs) have recently gained much attention due to a wide range of possible biomedical applications, including in vitro and in vivo imaging, drug delivery, photothermal therapy, etc. [1–4]. For therapeutic and diagnostic purposes, AuNPs are mainly administered intravenously and after entering the blood, the nanomaterial surface is instantly coated with plasma proteins. The protein corona provides AuNPs with a biological identity which is different from the original, engineered one and has a consequence for cellular uptake, accumulation, degradation and elimination of nanomaterial from the body [5]. Moreover, protein adsorption onto the nanoparticle surface induces the protein conformational transformations that can influence the biological reactivity of nanomaterial [6, 7]. Therefore, examining the relation between nanomaterial properties (such as the shape, size, type of terminal groups on its surface, etc.) and a tendency to adsorb specific plasma proteins may lead to a better understanding of nanoparticle behavior in vivo. The obtained information can be valuable in terms of developing new theranostic nanomaterials that could reach the desired region in the body without major side effects.

Analytical techniques suitable for characterization of the protein corona can be subdivided into two basic categories: (1) direct methods usually applied to analyze the proteins adsorbed on the nanomaterial surface, e.g., circular dichroism [8] or Fourier transformed infrared spectroscopy [9], and (2) indirect methods with the help of which changes in the nanoparticle properties are scrutinized (e.g. dynamic light scattering [10] or differential centrifugal sedimentation [11]). It should be emphasized that using some indirect techniques, such as fluorescence correlation spectroscopy [12] or nanoparticle tracking and analysis by light scattering [13], makes it possible to examine the protein corona in situ. On the other hand, techniques such as ESI–MS [14–16] and transmission electron microscopy [17] require that the excess of protein is removed prior to or during analysis. Application of the separation-based techniques, such as liquid/nanoliquid chromatography, capillary electrophoresis (CE) and gel electrophoresis, in investigations of the protein-bound nanoparticles has received a great deal of recent research trials [18–32]. Among these methods, CE has been most widely used to explore nano–bio interactions, as follows from a recent literature examination by Aleksenko et al. [33]. In that review, the authors discussed merits and present shortcomings of the CE method in the area, placing special focus on contributions based on using inductively coupled plasma mass spectrometry (ICP-MS) detection. It was concluded that recent advent of ICP-MS made CE a versatile speciation tool for biomedical studies of nanomaterials containing metals. In our original research, we utilized CE–ICP-MS specifically to explore compositional changes of metal-based nanoparticles in human serum environment [34, 35]. However, while the formation of distinct protein conjugates was recorded and characterized both kinetically and thermodynamically, in most of cases they remained unidentified. To cope with this challenge, it was deemed essential to portray the conjugates of the same AuNPs with individual serum proteins, as well as their physiologically-like mixtures, under identical sample preparation/separation conditions. This is the main objective of the present work.

## Materials and Methods

### Chemicals

Suspensions of spherical AuNPs (sAuNPs; 5, 10, 20, and 50 nm in diameter, functionalized with and stabilized by citric acid) were purchased from British Biocell International (Cardiff, UK) and stored in darkness at 4 °C. Rod-shaped AuNPs (rAuNPs) (6 × 29 nm), functionalized with polyethylene glycol and further modified by introducing amino- or carboxy-groups, were synthesized at the University of Florence. Human serum albumin, transferrin, apo-transferrin, and holo-transferrin, as well as chemicals used to prepare buffer solutions (Na_2_HPO_4_, NaH_2_PO_4_, 4-(2-hydroxyethyl)piperazine-1-ethanesulfonic acid or HEPES, piperazine-*N*,*N*′-bis(2-ethanesulfonic acid, PIPES), were the products of Sigma-Aldrich (St. Louis, MO, USA).

### Instrumentation

Analyses were performed on a HP^3D^CE system (Agilent Technologies, Waldbronn, Germany) coupled to a 7500a ICP mass spectrometer (Agilent Technologies, Tokyo, Japan) via a microconcentric CEI-100 nebulizer (CETAC, Omaha, NE, USA) and a custom-machined low-dead-volume conical spray chamber. A cross-piece was used to merge the sheath liquid flow. Polyimide-coated fused-silica capillaries (i.d. 75 mm; o.d. 375 mm; length 70 cm) were purchased from CM Scientific Ltd. (Silsden, UK). The^197^Au, ^34^S, and ^57^Fe isotopes were monitored to observe the speciation changes upon binding of nanoparticles with proteins. The signal of ^72^Ge was recorded to control the stability of CE flow and hyphenation performance as well as the efficiency of nebulization. Instrumental control and data analysis were performed using Agilent ChemStation software. Procedures for capillary initialization and rinsing before and between analyses are described elsewhere [34, 35]. Optimum operational parameters of CE–ICP-MS are presented in Table 1.

**Table 1:** CE–ICP-MS operational parameters

CE system	
Capillary	Fused silica capillary, I.D. 75 μm, O.D. 375 μm, length 70 cm
Capillary electrolyte	HEPES 40 mM, pH 7.4 (sAuNPs) PIPES 10 mM, pH 7.4 (rAuNPs)
Voltage	15 kV (sAuNPs) 12 or 20 kV (rAuNPs)
Temperature	37 °C
Sample injection	Hydrodynamic, 100 mbar × s
ICP-MS system	
RF power	1320–1400 W
Sample depth	6.7–7.0 mm
Plasma gas	15.0 L min^−1^
Nebulizer gas flow	1.0 L min^−1^
Monitored isotopes	^197^Au, ^72^Ge, ^57^Fe, ^34^S

### Sample Preparation and Analysis

Protein conjugation of AuNPs was performed as described previously [34, 35]. Briefly, the AuNPs diluted in 10 mM phosphate buffer (pH 7.4) containing 100 mM NaCl (final gold concentrations 9.5 and 4.3 or 1.7 mg L^−1^ for sAuNPs and rAuNPs, respectively) were added to an individual protein solution in simulated physiological buffer (10 mM phosphate buffer, pH 7.4, 100 mM NaCl) and the mixture was incubated at 37 °C for a desired period. Final concentrations of albumin and transferrin were 19 and 3 mg L^−1^ (sAuNPs samples) or 1.5 and 0.1 g L^−1^ (rAuNPs samples), respectively. Optimized CE conditions (see Table 1 and Refs. [34, 35] for more detail) ensured satisfactory separation efficiency and quantitative elution of AuNPs and their protein conjugates. Also importantly given the scope of this study, the repeatability of migration times was high enough (≤7% RSD; see ESM Table S-1) for a successful identification of nanoconjugates by this migration parameter. ICP-MS conditions were optimized using a tune-up solution containing 10 µg L^−1^ Li, Y, Ce, and Bi to obtain the highest signals for yttrium and bismuth and the lowest level of polyatomic, oxide and doubly charged ions.

## Results and Discussion

### Interaction of Nanoparticles with Albumin

**Fig. 1:**
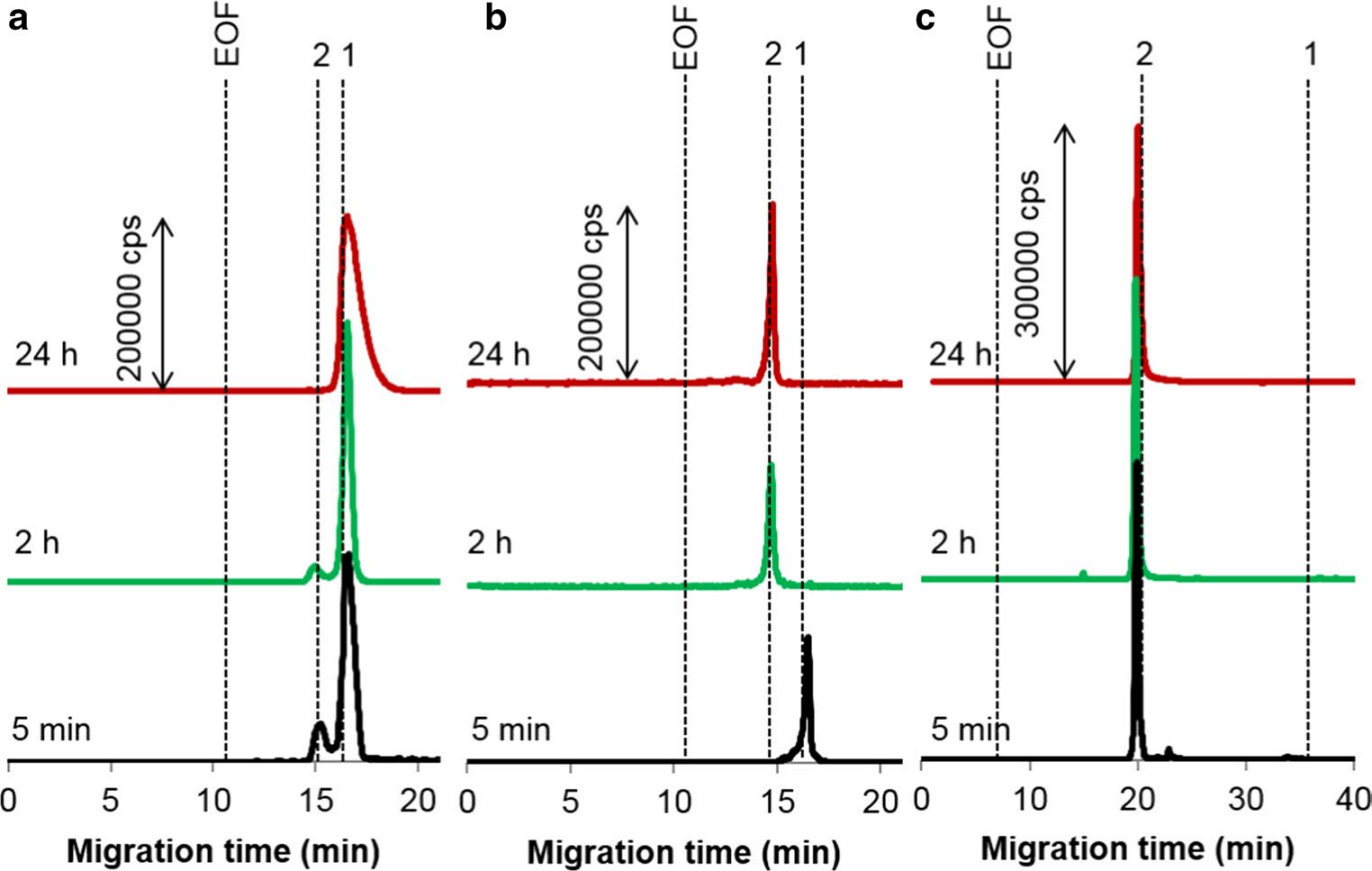
^197^Au electropherograms illustrating interaction between rAuNPs and albumin under simulated extracellular conditions. Particles (molar ratio to the protein): **a** carboxy-rAuNPs (1:1); **b** carboxy-rAuNPs (1:2.5); **c** amino-rAuNPs (1:1). Concentrations: albumin 1.5 g L^−1^; gold (**a**) and **c** 4.3 mg L^−1^, **b** 1.7 mg L^−1^. Peak assignment: *1* free rAuNPs, *2* albumin conjugate, *EOF* electroosmotic flow. Other conditions, see Table 1

Since the conjugation of sAuNPs with albumin, the most abundant plasma protein, has been in-depth examined earlier [34, 36–38], rAuNPs of different surface functionalization were the target of these binding experiments. As can be expected, the type of terminal groups regulates the rate of the corona formation (cf. traces a and c in Fig. 1), nanoparticles containing amino groups having a higher reactivity toward albumin than carboxyl-modified analogues. In the latter case, albumin molecules initially surround not more than 20% of nanoparticles and upon further incubation the conjugates are underwent decomposition (see also ESM Fig. S-1). Finally, after 24 h, carboxy-rAuNPs return into initial, protein-free form. Increasing the protein-to-metal ratio breaks down this tendency and after 2 h, all rod-shaped nanoparticles are converted in the conjugated form (Fig. 1b). This finding confirms that the applied dose of nanomaterial is a crucial factor governing the trafficking of AuNPs to the targets.

**Fig. 2:**
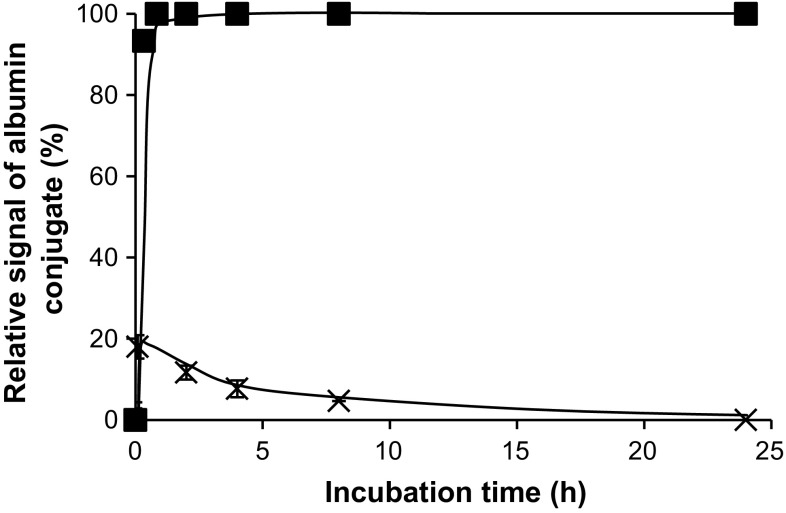
Binding of 5-nm sAuNPs (*squares*) and carboxy-rAuNPs (*crosses*) to albumin as a function of time. Gold concentration, 9.5 and 4.3 mg L^−1^, respectively

It is important to note that our observations are mostly in accord with the data of Scaletti et al. [39], who found out that the reaction of amino-functionalized rAuNPs with BSA led to greater changes in the intensity of plasmonic extinction band than in case of the carboxylated particles. Using BSA instead of HSA, however, is always an approximation as these proteins are compositionally homogeneous only for 76% [40]. Also interesting was to compare AuNPs of both types of shape at their doses and molar ratios to albumin mimicking circumstances to be encountered in blood (Fig. 2). From this figure, it is obvious that the albumin-binding affinity of sAuNPs is much higher, likely, on the account of more accessible metal centers.

### Interaction of Nanoparticles with Transferrin

**Fig. 3:**
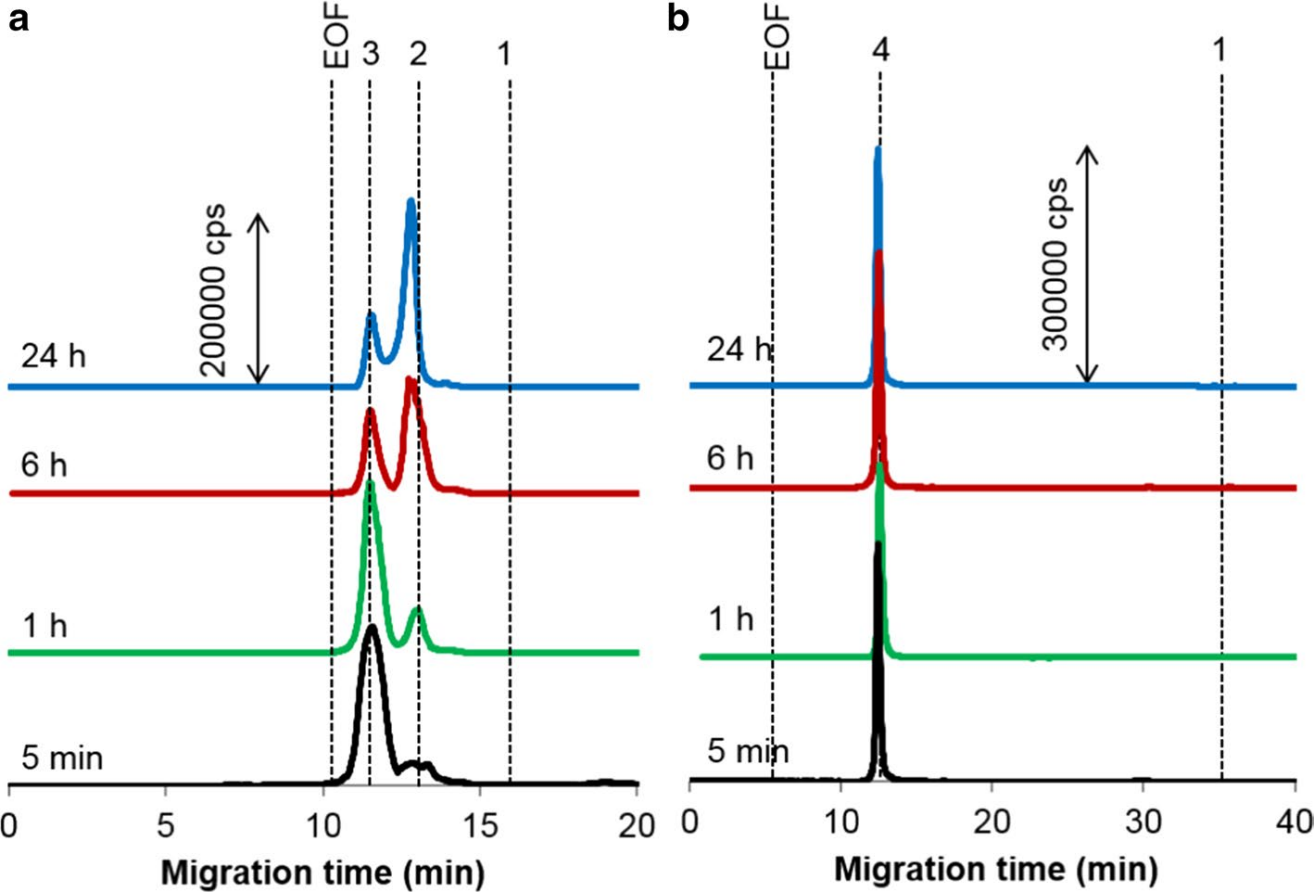
^197^Au electropherograms of the conjugates of **a** carboxy- and **b** amino-rAuNPs with transferrin. Concentrations: transferrin, 0.1 g L^−1^; gold, 4.3 mg L^−1^ after various incubation times. Peaks: *1* free particles, *2* conjugate with apo-transferrin, *3* conjugate with holo-transferrin, *4* conjugate with transferrin. Other conditions, see Table 1

**Fig. 4:**
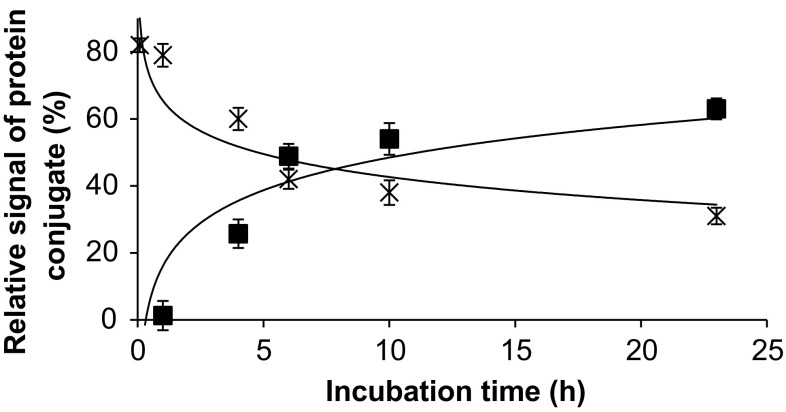
Interconversion of carboxy-rAuNPs conjugates with holo- (*crosses*) and apo-transferrin (*squares*). Gold concentration, 4.3 mg L^−1^. For regression parameters, see ESM Table S-2

For sAuNPs, binding to transferrin also reaches equilibrium fast (in ca. 5 min), though most of the particles remain uncovered with the protein such as 90% of 50-nm particles [34]. In contrast, rAuNPs became fully conjugated after the same period of incubation (Fig. 3), regardless of the type of functionalization. This implies that for nanoparticles with high affinity to the protein, the nature of terminal groups has lesser influence on the rate of the corona formation. As can also be seen in Fig. 3, for carboxy-rAuNPs, displaying higher electrophoretic mobility than amino-rAuNPs, the signals corresponding to the conjugates of apo- (iron-void form) and holo-transferrin (iron-saturated form) are well resolved. This allowed us to follow the time-dependent changes in relative content of both types of transferrin conjugates, as shown in Fig. 4. Remarkably, after equilibrium is attained (at about 24 h), the shares the transferrin forms take in forming the corona correspond to their physiological ratio, i.e., 70% of apo- and 30% of holo-transferrin (see also below). This observation was verified by analyzing carboxy-rAuNPs incubated for 24 h with a mixture of two protein standards of the same concentration ratio (Fig. S-2; compare to Fig. 3a, upper trace).

### Interaction with the Physiological Mixture of Albumin and Transferrin

**Fig. 5:**
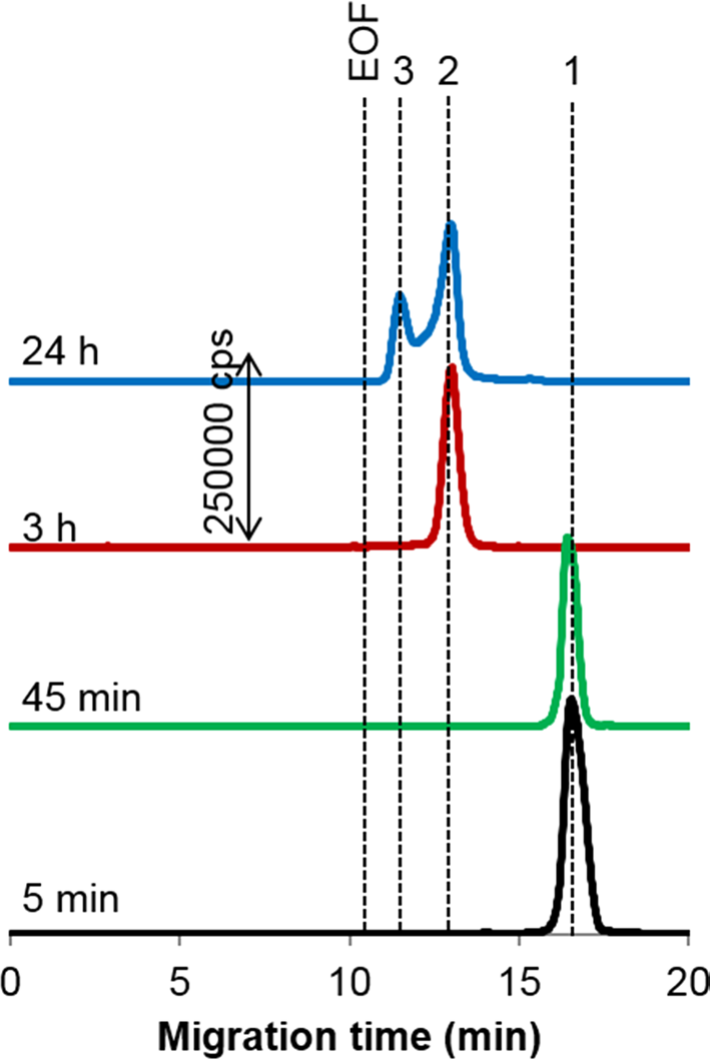
^197^Au electropherograms showing the proteinization of carboxy-rAuNPs (4.3 mg L^−1^ Au) in the mixture of human transferrin (0.1 g L^−1^) and albumin (1.5 g L^−1^) as a function of the incubation time. Peak assignment as in Fig. 3. Other conditions, see Table 1

In human blood, the concentration of albumin falls in the range from 35 to 50 g L^−1^, while the transferrin content is about 15-fold lower (2–4 g L^−1^) [41, 42]. When sAuNPs were subject to treatment with a mixture of these proteins simulating the physiological situation, only a single signal belonging to the albumin conjugate was recorded, no matter what the particle size was or their dose tested (data not shown). In each case explored, a complete conversion of sAuNPs into the albumin-bound form was faster than 2 min and the protein corona was stable even after 24 h of observation. Quite another binding behavior was characteristic to rAuNPs that demonstrate much slower reactivity toward the proteins under scrutiny. Figure 5 shows no peaks recorded before 2 h, and formed the first was the conjugate of apo-transferrin. Afterward, a two-protein binding scenario was observed, with holo-transferrin contributing at maximum 28% in the equilibrium proteinization. It should be underscored again that the equilibrium state is like that found out when transferrin was the only binding partner or the physiological mixture of two transferrin forms was applied (see above). The fact that albumin does not take part in the speciation changes in the presence of transferrin is quite understandable, considering poor albumin affinity of this sort of nanomaterial (see Fig. 1a).

## Conclusion

In this work, CE equipped with ICP-MS detection was used as an analytical system proved fitting perfectly to carry out metallic nanoparticle bioanalyses under physiological conditions. Alterations of AuNPs in the presence of serum proteins and their mixtures was shown to depend on the nanoparticle geometry and size, surface functionalization and applied dose (nanoparticle-to-protein molar ratio), as well as time for which AuNPs reside in a proteinaceous setting. Perhaps most interestingly, the protein-binding kinetics for differently shaped AuNPs was for the first time compared within a single CE–ICP-MS study. Provided that the results on the characterization of individual nano-protein conjugates would be supported by further studies, using a molecular-type or/and high-resolution ICP-MS detector, the impending task of deciphering the protein-mediated transformations of AuNPs on the way to the target could be completed.

## Electronic supplementary material

Below is the link to the electronic supplementary material.

Supplementary material 1 (DOCX 33 kb)
