# Supplementary material for: CE Separation and ICP-MS Detection of Gold Nanoparticles and Their Protein Conjugates
Source: Chromatographia. 2017 Aug 24;80(11):1695–700. doi: 10.1007/s10337-017-3387-y (PMC5681605; doi:10.1007/s10337-017-3387-y)
Supplement: Supplementary file 1 — Supplementary material 1 (DOCX 33 kb) [file 10337_2017_3387_MOESM1_ESM.docx]

**CE Separation and ICP-MS Detection of Gold Nanoparticles and Their Protein Conjugates**

**Joanna Legat • Magdalena Matczuk • Andrei Timerbaev • Maciej Jarosz**

**Supporting Material**

**Table S-1** Repeatability of migration times of rAuNP–protein conjugates

| Conjugate^a^ | | RSD of migration time (%)^b^ |
| --- | --- | --- |
| Albumin | Carboxy-rAuNPs  Amino-rAuNPs | 6.4  6.9 |
| Apo-transferrin | Carboxy-rAuNPs  Amino-rAuNPs | 4.2  5.7 |
| Holo-transferrin | Carboxy-rAuNPs  Amino-rAuNPs | 7.5  6.0 |

^a^Formed at protein concentrations corresponding to the levels in 30-times diluted human serum. ^b^*n* = 6.

**Table S-2** Parameters of logarithmic regression for the kinetics of interconversion of carboxy-rAuNP conjugates

| Conjugate | Equation | Regression coefficient (*r*²) | Mean percentage error  (%) |
| --- | --- | --- | --- |
| Apo-transferrin | y = 10.95*ln*(x) + 32.48 | 0.909 | 19.0 |
| Holo-transferrin | y = –9.91*ln*(x) + 65.46 | 0.901 | 12.9 |

**Fig. S-1** Time-dependent changes in relative signal for free carboxy-rAuNPs (crosses) and their albumin conjugate (squares). Gold concentration, 4.3 mg L^–1^ (molar ratio of gold to albumin 1:1).


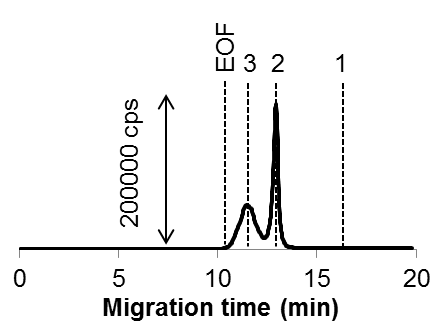


**Fig. S-2** ^197^Au electropherogram of a mixture, containing 30% holo- and 70% apo-transferrin (total protein concentration, 0.1 g L^–1^) interacted with carboxy-rAuNPs (4.3 mg L^–1^Au) for 24 h. Peaks: 1 – free particles; 2 – apo-transferrin conjugate; 3 – holo-transferrin conjugate. Other conditions, see Table 1.
